# Shewanella algae Bacteremia and Septic Shock Secondary to Necrotizing Fasciitis: A Case Report

**DOI:** 10.7759/cureus.86259

**Published:** 2025-06-18

**Authors:** Gerald Tse, Natesha E Angullia

**Affiliations:** 1 Department of Anaesthesiology, Singapore General Hospital, Singapore, SGP

**Keywords:** immunocompromised host, necrotising fasciitis, opportunistic infection, post-bariatric surgery, septic shock, shewanella algae

## Abstract

*Shewanella algae *(*S. algae*), a marine Gram-negative bacillus, is an emerging opportunistic pathogen capable of causing infections in humans. While mostly associated with mild skin and soft tissue infections, severe invasive infections, such as bacteremia and septic shock, are rare but increasingly reported. We present a case of fatal *S. algae* bacteremia and septic shock secondary to necrotizing fasciitis in a patient with significant comorbidities, including recent bariatric surgery, rapid weight loss, and possible malnutrition, highlighting the role of host factors in fulminant disease progression.

## Introduction

*Shewanella algae* (*S. algae*) is a Gram-negative, facultatively anaerobic bacillus [1] increasingly recognized as a human pathogen with a broad spectrum of clinical manifestations. Although traditionally associated with marine environments [2], infections with *S. algae* are now being reported globally, including in patients without direct aquatic exposure, as in our case [3].

Necrotizing fasciitis due to *S. algae* is rare but carries high morbidity and mortality [4]. We present a fatal case of *S. algae *necrotizing fasciitis and septic shock in a patient with class III obesity, recent bariatric surgery, and no documented marine exposure, exploring the role of rapid weight loss and nutritional immune impairment in disease susceptibility.

## Case presentation

A 60-year-old female presented to the emergency department with a one-day history of right lower limb pain and swelling. There were no antecedent falls or trauma. Her past medical history was significant for hypertension, hyperlipidemia, obstructive sleep apnea, chronic venous insufficiency, and class III obesity with a body mass index (BMI) of 45. Notably, she had a gastric bypass surgery 10 months prior, during which she had a BMI of 63. She had lost 47 kg in 10 months.

On presentation, she was febrile (39.0°C), hypotensive (blood pressure 69/50 mmHg), tachycardic (heart rate 120 beats per minute), and tachypneic (respiratory rate 40). Physical examination revealed tense bullae over her right calf associated with warmth and mild crepitus. This was associated with bilateral lower limb hyperpigmentation and pitting edema.

**Figure 1:**
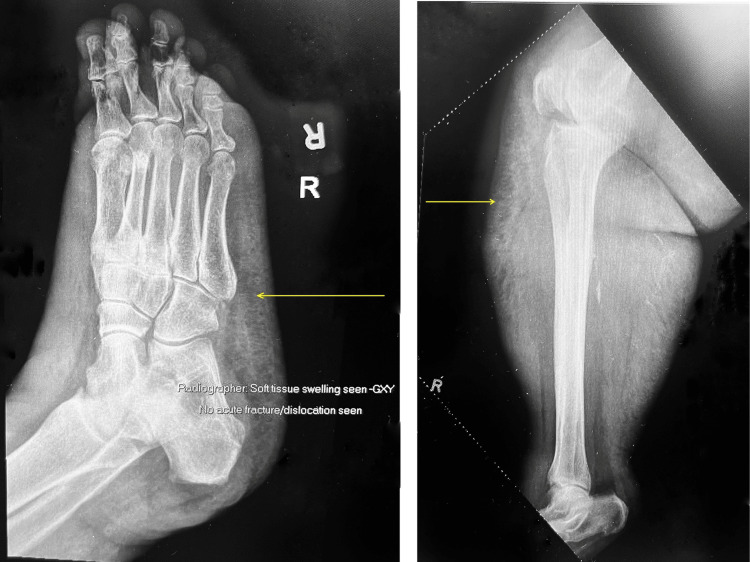
Right lower limb X-rays Subcutaneous gas and soft tissue swelling are marked by the yellow arrows. These findings support the diagnosis of necrotizing fasciitis.

Laboratory investigations revealed leukopenia (WBC 3.3 × 10⁹/L) and elevated C-reactive protein (30.6 mg/L). This was associated with elevated creatinine (141 μmol/L), elevated bilirubin (38.3 μmol/L), thrombocytopenia (platelets 93 × 10⁹/L), coagulopathy (prothrombin time (PT) 26.6 seconds, international normalized ratio (INR) 2.55, partial thromboplastin time (PTT) 68.1 seconds), and metabolic acidosis (pH 7.27, serum bicarbonate 7.0 mmol/L, anion gap 28 mmol/L) with elevated lactate levels (18.4 mmol/L). The key laboratory investigations on admission are shown in Table 1. X-rays of her right lower limb revealed gas streaks (Figure 1).

**Table 1:** Key laboratory investigations on admission

Laboratory investigation	Result	Reference range
White blood cell count	3.3 × 10⁹/L	4.0-10.0 × 10⁹/L
Platelet count	93 × 10⁹/L	140-440 × 10⁹/L
Hemoglobin	10.3 g/dL	14.0-18.0 g/dL
Sodium	138 mmol/L	135-145 mmol/L
Potassium	3.7 mmol/L	3.5-5.0 mmol/L
Chloride	103 mmol/L	100-107 mmol/L
Bicarbonate	7.0 mmol/L	19.0-29.0 mmol/L
Creatinine	141 μmol/L	54-101 μmol/L
Lactate	18.4 mmol/L	0.5-2.2 mmol/L
Total bilirubin	38.3 μmol/L	7-32 μmol/L
C-reactive protein	30.6 mg/L	0.2-9.1 mg/L
Prothrombin time (PT)	26.6 seconds	9.7-11.6 seconds
International normalized ratio (INR)	2.55	-
Partial thromboplastin time (PTT)	68.1 seconds	24.2-35.5 seconds
pH	7.27 mmHg	7.35-7.45 mmHg
pCO_2_	<15.0 mmHg	35.0-45.0 mmHg
pO_2_	133.5 mmHg	75.0-100.0 mmHg
Base excess	-18.6 mmol/L	-2.0-2.0 mmol/L

Given the clinical suspicion for necrotizing fasciitis and septic shock, the patient was started on empirical antibiotics (ceftazidime, clindamycin, and benzylpenicillin) in accordance with our Institution's empirical antibiotic guidelines for necrotizing soft tissue infections, along with fluid resuscitation. She was then transferred to the operating theatre for urgent surgical debridement. Prior to induction of general anesthesia, 1500 mL of crystalloids and 500 mL of 5% albumin were administered. An intra-arterial line was inserted into the left radial artery. Noradrenaline was initially started via a peripheral intravenous line in the cubital fossa at a dose of 0.2 mcg/kg/minute. This was done as a central venous catheter could not be safely sited as the patient was in extremis. General anesthesia was then induced with intravenous midazolam 3 mg, ketamine 30 mg, fentanyl 50 mcg, and suxamethonium 150 mg, and the patient was intubated. Intravenous hydrocortisone 100 mg was administered to support hemodynamic stability by enhancing vascular responsiveness to catecholamines and to address possible relative adrenal insufficiency, in accordance with the Surviving Sepsis Campaign guidelines. Following induction and tracheal intubation, a central venous catheter was then placed in the right internal jugular vein, and the noradrenaline infusion was continued, requiring a maximum dose of 0.2 mcg/kg/minute to target a mean arterial pressure of greater than 65. Anesthesia was maintained with sevoflurane, and atracurium was used for neuromuscular blockade. Depth of anesthesia was titrated with the use of the Masimo SedLine (Masimo, Irvine, CA). The patient was ventilated on volume-controlled ventilation with an inspired oxygen fraction of 0.4. Intraoperatively, extensive necrosis of subcutaneous tissue and fascia was confirmed, consistent with necrotizing fasciitis.

Post-operatively, the patient was admitted to the intensive care unit (ICU) for hemodynamic support and mechanical ventilation. Blood cultures subsequently grew *S. algae* that was pan-sensitive (sensitive to ceftazidime, ciprofloxacin, cotrimoxazole, gentamicin, and meropenem). Intra-operative tissue cultures also grew *S. algae* that was pan-sensitive.

Despite aggressive surgical and medical management, the patient’s condition continued to deteriorate, with progressive multiorgan failure including renal and liver failure, as well as disseminated intravascular coagulation (DIC). Vasopressin was initiated in addition to noradrenaline to support her worsening hemodynamic status. However, continuous renal replacement therapy could not be safely initiated due to persistent hemodynamic instability. The patient died approximately 24 hours after her initial presentation.

## Discussion

*S. algae* infections can manifest as skin and soft tissue infections (SSTIs), bacteremia, hepatobiliary infections, ear infections, and, less commonly, central nervous system involvement [2,3,5,6]. SSTIs, including cellulitis, necrotizing fasciitis, and abscesses, are among the most frequently reported presentations. In a case series of 128 Shewanellainfections, 20.1% involved skin and soft tissue [5], and among 273 global cases reviewed, SSTIs and bloodstream infections were the most common clinical syndromes [3].

Our case of necrotizing fasciitis complicated by bacteremia and septic shock underscores the severe potential of *S. algae *infections, particularly in patients with comorbidities. The literature identifies several predisposing factors: chronic liver disease [5,7,8], malignancy [4,5], diabetes mellitus [5], renal impairment [9,10], peripheral vascular disease [5], and immunocompromised states [6]. In this case, class III obesity, chronic venous insufficiency, and possible post-bariatric surgery malnutrition likely contributed to susceptibility and severity. Rapid weight loss following bariatric surgery can result in protein-calorie malnutrition, micronutrient deficiencies (zinc, selenium, and iron), and immune dysfunction, increasing vulnerability to opportunistic infections [11-13]. These nutritional deficits can be due to both pre-existing deficiencies in obese patients and the malabsorptive or restrictive nature of bariatric procedures, which reduce nutrient intake and absorption. Although bariatric surgery may lead to changes in immune function [11-14], it remains uncertain whether these changes result in clinically significant immune suppression or increased susceptibility to opportunistic infections. Given the aggressive course here, it is plausible that a post-surgical immunocompromised state, compounded by baseline vascular insufficiency, allowed for fulminant progression of the infection.

The pathogenesis of invasive *S. algae* infection remains incompletely understood but is thought to involve multiple virulence factors, including hemolysins, exotoxins, and biofilm formation, facilitating tissue invasion and immune evasion [3,15,16]. Our patient’s rapid deterioration and multiorgan failure mirror other fulminant cases reported in both immunocompromised and postoperative patients [6-9].

Antimicrobial susceptibility patterns remain variable. In general, *S. algae* remain susceptible to third- and fourth-generation cephalosporins, aminoglycosides, and fluoroquinolones [2,5]. However, increasing resistance to carbapenems has been documented [4,5,17]. In our case, ceftazidime, ciprofloxacin, and benzylpenicillin were started empirically. This is in line with our Institution’s empirical antibiotic guidelines for the treatment of necrotizing fasciitis. It remains an appropriate empirical choice, though susceptibility testing is essential given emerging resistance patterns [4,17,18].

Prompt diagnosis and source control in the form of aggressive surgical management are critical for *S. algae* necrotizing fasciitis, as delayed intervention correlates with increased mortality. Despite timely debridement and intensive care in our case, the infection progressed rapidly, reflecting the fulminant potential of *S. algae* in vulnerable hosts.

## Conclusions

This case highlights the importance of recognizing *S. algae* as a potential pathogen in severe SSTIs. Clinicians should maintain a high index of suspicion, especially in patients with underlying risk factors that predispose them to opportunistic infections.

It also underscores the need for further research into the immunological consequences of bariatric surgery and rapid weight loss, as these procedures become increasingly common. A better understanding of post-surgical nutritional and immune changes may help guide perioperative care and infection prevention.
